# Surpass Evolve Flow Diverter for the Treatment of Intracranial Aneurysm: A Systematic Review

**DOI:** 10.3390/brainsci12060810

**Published:** 2022-06-20

**Authors:** Rania Issa, Zahrah Al-Homedi, Dawood Hasan Syed, Waseem Aziz, Basem Al-Omari

**Affiliations:** 1 College of Medicine and Health Sciences, Khalifa University, Abu Dhabi P.O. Box 127788, United Arab Emirates; 100052941@ku.ac.ae (R.I.); 100049881@ku.ac.ae (Z.A.-H.); 100052900@ku.ac.ae (D.H.S.); 2 Department of Neurosurgery, Sheikh Shakhbout Medical City, Abu Dhabi P.O. Box 11001, United Arab Emirates; wassim.almawi@adu.ac.ae; 3 Department of Neurosurgery, Alexandria University School of Medicine, Alexandria 21526, Egypt; 4 Department of Epidemiology and Population Health, College of Medicine and Health Sciences, Khalifa University, Abu Dhabi P.O. Box 127788, United Arab Emirates; 5 KU Research and Data Intelligence Support Center (RDISC) AW 8474000331, Khalifa University of Science and Technology, Abu Dhabi P.O. Box 127788, United Arab Emirates

**Keywords:** surpass evolve, flow diverter, Stryker, intracranial aneurysm, systematic review

## Abstract

Purpose: This systematic review aims to summarize the evidence investigating the effectiveness and safety of the Surpass Evolve-Flow Diverter (SE-FD) to treat brain aneurysms. Method: We searched MEDLINE, EMBASE, CINAHL, Web of Science, and Cochrane Library from January 2019 to 29 March 2022. Terms related to the “intracranial aneurysm” and “surpass evolve flow diverter” concepts were used to search the databases; Medical Subject Headings (MeSH) and reference hand search were also utilized. Results: The searches primarily identified 1586 documents. A total of five studies (four case series and one cohort) were included in this review. In the included studies, 192 (74 male and 118 females) patients with 198 aneurysms were involved. In total, 153 SE-FDs were used to treat 145 aneurysms. Complete occlusion was achieved in 69/145 (48%) cases and near-complete occlusion in 24/145 (17%) cases from aneurysms treated with SE-FD. Reported postoperative complications included stent thrombosis (*n* = 4 patients), hemorrhage (*n* = 5 patients), ischemia (*n* = 9 patients), and neurological complications (*n* = 12 patients). In total, four deaths were reported with only one related to the SE-FD procedure. Conclusion: The results of this review are based on observational data, due to the absence of clinical trials. The findings of the included studies suggest that the effectiveness of the SE-FD procedure is lower than previous FDs but the safety is similar. The included studies also suggested that SE-FD has navigability and resistance to twisting, which makes the procedure an easier method to treat aneurysms that are proximal and distal to the circle of Willis deployment. This review highlights the urgency to conduct clinical trials to confirm these suggestions.

## 1. Background

A brain aneurysm is a critical condition that mostly affects young adults and may cause life-threatening complications such as brain hemorrhage [1,2]. Furthermore, patients with untreated unruptured aneurysms have 50% excess long-term mortality compared with the general population [3]. One of the most innovative treatment options for aneurysms is the use of a flow diverting stent (FDS) [4]. FDS was first introduced in 2007 to treat a wide range of aneurysms such as large, giant, and wide-neck types [5,6]. The FDS mechanism works by redirecting the flow of blood within the parent artery, moving the flow away from the aneurysm [7], and promoting thrombosis which subsequently leads to aneurysm occlusion [8]. A recent systematic review of 26 studies of FDS retreatment between the year 2000 and 2021 demonstrated that FDS is an effective retreatment strategy for intracranial aneurysms, except in patients with non-saccular aneurysms, and recommended FDS as a first-line option for patients with recurrent intracranial aneurysm [9].

The main characteristics of FDS are represented in porosity, pore density, and metal coverage [8]. Porosity is defined as the proportion of the surface area without metal coverage over the total surface area, metal coverage is the percentage of the area that is covered by the flow diverter, and pore density is the number of pores per unit area [8,10,11]. A lower porosity contributes to a faster occlusion of the aneurysm [12]. Furthermore, increasing the metal coverage of the FDS leads to lowering the porosity [6]. Therefore, porosity, pore density, and metal coverage all contribute to improving the quality of FDS which leads to a better occlusion rate [8,12].

The first FDS (pipeline and SILK stents) used 48 wires. Moreover, there was a thought of increasing the number of wires to increase the metal coverage and reduce the porosity [13]. Therefore, Surpass Streamline (SS-FD) was introduced to containing a total of 48 to 96 wires [14]. This change caused a challenge in the navigation and deployment of the FDS [5]. Thus, Surpass Evolve-Flow Diverter (SE-FD) was introduced in 2019 as an updated version of the previous surpass model [15]. To optimize and maintain the flow diversion effect and maintain effective navigation and deployment, SE-FD uses 64 wires [6,16]. It is believed that the introduction of SE-FD may effectively maintain a therapeutic porosity, pore density, and metal coverage. Furthermore, SE-FD uses a small number of wires in comparison to SS-FD, which theoretically decreases the risk of perforator infarctions [17]. However, this hypothesis of reduction of risks of perforations is still to be tested with human subjects in clinical trials. Therefore, the aim of this systematic review is to retrieve and summarize suitable studies that investigated the effectiveness and safety of SE-FDs to treat brain aneurysms.

## 2. Materials and Methods

### 2.1. Search Strategy

A systematic reviewer (BA) developed a comprehensive search strategy. PubMed (Medline), EMBASE, CINAHL, Web of Science, and Cochrane Library were electronically searched from January 2019 to 29 March 2022. The search was limited to 2019, which is the year when SE-FD was manufactured. Medical Subject Headings (MeSH) and search terms were used to interrogate the databases. Two concepts related to “intracranial aneurysm” and “surpass evolve flow diverter” were used to search the databases (for search terms and a search example see Supplementary S1). No restriction on publication language was applied. Additionally, a hand search of the reference list of the published articles was also used to identify additional publications.

The protocol for this review was registered with the International Prospective Register of Systematic Reviews (PROSPERO) under registration number CRD42022298038 (available from https://www.crd.york.ac.uk/prospero/display_record.php?RecordID=298038) (accessed on 21 May 2022)). Reporting of this systematic review was guided by the Preferred Reporting Items for Systematic Reviews and Meta-Analysis (PRISMA) [18].

### 2.2. Criteria for Considering Studies for the Review

Any study designs examining the use of SE-FD to treat adult patients (over the age of 18 years old) with intracranial aneurysms were considered for inclusion. Studies were excluded if the results did not report SE-FD outcomes or if the outcomes were for several FDS procedures combined.

### 2.3. Study Selection Process

All retrieved records were imported to Covidence web-based application and duplicate records were removed. The first step of study selection was screening the titles and abstracts of all records, and then the full text of relevant papers was screened for eligibility for inclusion in the review. At least two reviewers conducted the study selection process independently. The reviewers discussed any disagreement in the first instance then a third reviewer resolved any further disagreement.

### 2.4. Quality Assessment

Quality assessment of included studies was conducted independently by two reviewers using Murad and colleagues’ framework for appraising case series [19] and the critical appraisal skills program (CASP) tool for appraising cohort studies [20] (Supplementary S2). The stars rating was used to score the included studies. Each question in the quality assessment tool can be given a maximum of one star. The maximum number of stars that can be given to cohort studies is 12 and 8 stars are the maximum for case series.

### 2.5. Data Extraction

Two reviewers performed the data extraction independently. The reviewers discussed any disagreement in the first instance then a third reviewer resolved any further disagreement. The following data were extracted: research methods, country of study, population, sample size, No. of Aneurysms, No. of SE, participants’ age mean/range, participants’ gender, studies main aims, studies primary and secondary outcomes, studies’ conclusions, type of aneurysms included in the studies, type of aneurysms treated with SE-FDs, period of follow-up, description of SE-FDs procedure, perioperative and postoperative complications, aneurysms occlusion, clinical and radiological outcome, technical success, morbidity, and deaths. Contacting authors for any missing data was considered.

### 2.6. Data Analysis

A narrative synthesis of the included studies was conducted. Meta-analysis was considered but was not possible due to heterogeneity of the studies.

## 3. Results

### 3.1. Studies Identified

**Figure 1:**
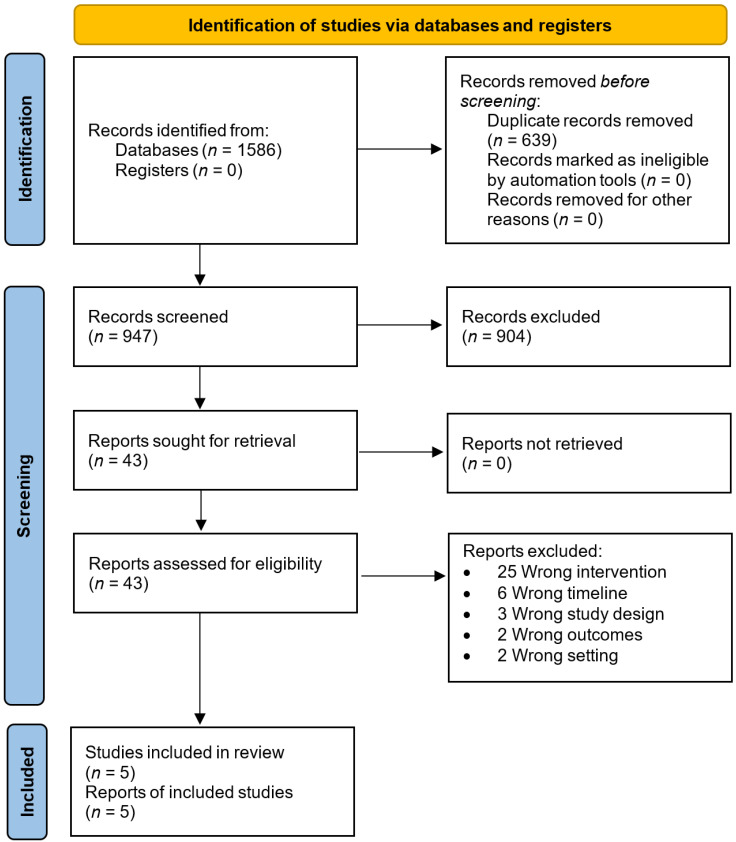
The PRISMA flow diagram of studies in the review.

The searches identified 1586 documents. After removing the duplicates, 947 documents remained. Subsequently, 904 documents were excluded based on title and abstract screening against the inclusion/exclusion criteria. A further 38 were excluded at the full-text screening for not meeting the inclusion criteria, leaving five studies for quality assessment [15,16,21,22,23] (see Figure 1 for PRISMA flow chart).

### 3.2. Quality Assessment

At least two reviewers assessed and reported the quality of all included studies. However, due to the very limited number of available studies, none were excluded at the quality assessment stage. A total of three case series scored 5/5 stars [15,22,23]. The remaining case series [16] scored 4/5 stars. Three authors (RI, ZA, and DS) reviewed the study and were unable to identify evidence within the paper that “other alternative causes that may explain the observation were ruled out”. The cohort study [21] scored 8/12 stars. The same three reviewers were unable to locate information suggesting that “the authors identified all-important confounding factors”, “taken account of the confounding factors in the design and/or analysis”, “followed up of subjects complete enough”, and “explained the implications of this study for practice” (see Table 1 for quality assessment scores).

### 3.3. Included Studies Characteristics

A total of three studies used the retrospective case series design [15,22,23], one used the prospective case series [16], and one used the retrospective cohort design [21]. Two studies were conducted in the Republic of Korea [21,22], two in Europe [15,23], and one in Canada [16]. The mean age for the participants ranged between 54.6 and 58 years. A total of 192 (74 male and 118 females) patients with 198 aneurysms were included in the five studies. A total of 153 SE-FDs were used across the five studies to treat 145 aneurysms (see Table 2 for the included studies’ characteristics).

### 3.4. Studies Aims, Outcomes, and Conclusions

All studies aimed to determine the safety and efficacy of using the new SE-FDs with two of the studies [21,22] comparing it with other FDs. The focus of all studies was on the clinical outcome of using SE-FDs, which includes aneurysm occlusion and clinical safety. Two studies [21,23] had more emphasis on the technical aspects of the flow diverter such as suboptimal wall opposition, intraprocedural or delayed stent migration, favorable navigation, and successful deployment of SE-FDs. Furthermore, radiographic follow-up was a major objective in the secondary outcome of the five studies. Overall, all studies concluded the ease and safe deployment of SE with promising occlusion rates (see Table 3).

### 3.5. Studies Type of Aneurysms, Follow-Up Period, and Description of the Procedure

A total of 198 aneurysms were included in the five studies, in which 145/198 (73%) in the anterior circulation and 53/198 (27%) in the posterior circulation. In terms of size; 74/198 (37%) aneurysms were small, 94/198 (47%) were large, and 30/198 (15%) were giant. Saccular aneurysms accounted for the majority of the cases 118/198 (60%), while non-saccular ones accounted for 80/198 (40%). The aneurysms treated with SE included 110/145 (76%) in the anterior circulation and 35/145 (24%) in the posterior circulation. Out of the total 145 aneurysms treated with SE, 74/145 (51%) were small, 57/145 (39%) were large, and 14/145 (10%) were giant. Saccular aneurysms accounted for 93/145 (64%) and 52/145 (36%) were non-saccular (see Table 4).

The period of follow-up ranged from five days to 16 months. All studies employed dual antiplatelet therapy (DAPT) using aspirin and clopidogrel for 3–14 days or bolus dose 1–2 days before surgery. In four of the included studies [15,21,22,23] the platelet function was assessed either using VerifyNow or Multiplate Analyzer. Based on the platelet function assessment, poor responders to clopidogrel were switched to ticlopidine or prasugrel. During the procedure, intravenous heparin was administered to maintain adequate clotting time. After the procedure, DAPT was continued for all patients for 3–6 months, then aspirin was prescribed for 6 months to life. Deployment of the FD was either through the femoral or radial artery. The type and number of FDs used were based on the operator’s discretion. Stent wall apposition was later detected using various methods including angiography, VasoCT, or DSA (see Table 4).

Two of the included studies [16,21] reported that the learning curve of the Pipeline Embolization Device (PED) is a predictive factor for procedure-related complication rate. Three studies [15,16,23] reported experiencing excellent navigability and resistance to twisting with SE-FDs. One study [16] suggested that a lower flow diverter profile and ease of deployment of SE-FDs might facilitate reaching aneurysms beyond the circle of Willis. A couple of years later, another study [15] reported treating three aneurysms beyond the circle of Willis with no major technical problem.

### 3.6. SE Complications and Outcomes

Of the total aneurysms treated with SE-FD, complete occlusion was achieved in 69/145 (48%) cases and near-complete occlusion was achieved in 24/145 (17%) cases after follow-up. Technical success was reported in all studies with a calculated percentage only in two studies as 90.3% [21] and 96% [23]. Four studies [15,16,22,23] reported the radiological outcomes using O’Kelly Marotta (OKM grade): D: 46, C:12, B:21, A:15. The remaining study [21] used magnetic resonance imaging (MRI) to measure the outer-to-outer diameter on pre-treatment and post-treatment at 5- and 30-days follow-up as well as digital subtraction angiography (DSA) and CT angiography at 30-days follow-up. All the other cases had good clinical outcomes and recovery, and long-term morbidity in two cases with modified Rankin Scale (mRS) of 4 and 2 reported. One death was reported in two studies [21,23], and two deaths in one study [15] (see Table 5 and Table 6).

Two out of the five included studies [21,23] reported complications perioperative to SE procedure, including incomplete wall opposition, stent migration, and acute in-stent thrombosis. Postoperatively only one study reported no complication [22], the remaining four studies [15,16,21,23] reported stent thrombosis in four patients, remote intraparenchymal hemorrhage (RIPH) complications in two patients, ischemic complications in nine patients, and neurological complications in 12 patients. Delayed stent migration and/ or aneurysm ruptures were reported in two studies [15,21] (see Table 6).

## 4. Discussion

The rate of complete occlusion in the five studies ranged from 35 to 75%. In comparison to SS-FD, the performance of SE-FD was lower in the majority of the included studies as the rate of the complete occlusion for SS-FD was 75% after 6 months [24] and 62.8% after 12 months [25,26]. The occlusion rate was also lower in all of the included studies than that in the PREMIER study (76.8%) using the Pipeline flow diverter, but the use of adjunctive coiling at 4%, and the SAFE study (73.3%) using FRED and FRED Jr. flow diverters with an adjunctive treatment in 25% of cases [27,28]. Four of the included studies [15,16,21,23] used SE-FD with adjunctive treatment and one study did not use adjunctive treatment and had a complete occlusion rate of 60% with 100% at follow-up [22]. A study evaluating the safety and effectiveness of six types of FDs (PEP, SILK, FRED, the p64 flow modulation device, SS-FD, and Derivo embolization device) reported complete occlusion of 49% at 3 months, 29% at 6 months, 12% at 12 months, and 1% at 18 months (91% in total) [29]. The complete occlusion rate for the total sample in this systematic review was 48% at a 4–7 months follow-up, which is lower than that reported in other FDs. A similar systematic review evaluating the prognosis and mortality rates for the Silk-FD reported complete occlusion of 80.4% for the 14 included studies combined [30].

In this review, three studies [15,16,23] reported that SE-FDs have excellent navigability and resistance to twisting. Furthermore, one of the included studies [16] suggested that the lower flow diverter profile and ease of deployment make SE-FD an appropriate device to treat aneurysms that are proximal and potentially distal to the circle of Willis. This effect was also evident in one of the studies [15] which treated three aneurysms beyond the circle of Willis with no major technical problem. The learning curve is a predictive factor for procedure-related complication rate, which was discussed for PED in two of the included studies [16,21]. Since SE-FD and PED are similar in their delivery system and deployment method, it is anticipated that they would have a similar learning curve but shorter than SS-FD.

Two studies reported one incomplete wall apposition in ICA due to the tortuous nature of the carotid siphon anatomy [21,23]. Both of these studies also reported one case of intraprocedural stent migration [21,23] which can usually occur during or after the procedure. The reported range of intraprocedural stent migration was from 2 to 45% in PED [31,32] and SS-FD [24]. Intraprocedural stent migration can be corrected with an additional stent immediately and delayed stent migration can lead to undesirable clinical outcomes in terms of delayed identification. Regarding SE-FD, only one study [21] reported one incident of delayed stent migration (3.2%) with a rate similar to the flow diversion PED from 2 to 5% [31,33,34].

Four studies reported the rate of in-stent thrombosis between 2% and 7% despite sufficient platelet inhibition [15,16,21,23]. This is consistent with the 6.9% previously reported risk of in-stent thrombosis [35]. Furthermore, one of the most dangerous complications of flow diverters is aneurysm rupture. There was no immediate aneurysm rupture reported in any of the included studies in this review. However, there were reports of delayed aneurysm rupture ranging from 0 to 3%; RIPH hemorrhage, from 0 to 7%; and ischemic stroke, from 0 to 4%. This is similar to a large clinical series of other FDs reporting the delayed aneurysm rupture from 0 to 4%; RIPH hemorrhage from 0 to 6%; and ischemic stroke from 1 to 10% [10,12,13,33,36,37]. A meta-analysis of FD suggested that the reason for SAH from the delayed ruptured aneurysm and ischemic stroke occurred in 4% and 6% of patients after flow diversion, with significantly higher rates among patients with large and giant aneurysms and aneurysms in the distal anterior and posterior location [5]. Another systematic review identified delayed ruptures in 81 aneurysms after treatment with FD, where giant aneurysms accounted for 46% of ruptures [38].

The rate of neurological complications was reported in nine patients out of the 145 who used the SE-FDs (6.2%) in the included studies with the majority of these patients having minor neurological complications without persistent morbidity. This rate is consistent with other FD neurological complications reported in SCENT and PUFS trials (8.3% and 5.6%, respectively) [25,39]. Only one patient had an mRS of 4 [16]. The reported death following SE was 2.8% (4/145) with only one related to the SE-FD procedure [15], which is consistent with the risk of death for previous FD [5,40,41,42].

### Strengths and Limitations

To the best of our knowledge, this is the first systematic review to summarize studies investigating the effectiveness of SE-FD to treat cranial aneurysms. A comprehensive search of the literature was conducted utilizing several databases. This review included five observational studies, due to the lack of clinical trials. Therefore, our results must be interpreted in line with the limitation of the research methods of the included studies. Meta-analysis was not possible due to heterogeneity of the studies, which is a common limitation in systematic reviews when quantitative summary of the studies is not possible due to limited evidence availability [43,44].

This review identifies three retrospective case series [15,22,23], one prospective case series [16], and one retrospective cohort design [21]. These methods of research are considered weaker forms of evidence than RCT. However, we consider the included papers to be of good quality research in relation to the conduct of these types of research methods. Although SE-FD has been used in the clinical setting for about three years [16,45], we were unable to identify any published clinical trial investigating the effectiveness of this type of FD. However, there is an ongoing clinical trial in the participants’ recruitment stage investigating the safety and effectiveness of SE-FD to treat unruptured, wide-neck intracranial aneurysms with anticipated completion in 2025 [46]. Conducting SE-FD clinical trials are delayed in comparison to previous FDs. SS-FD had already received FDA approval in 2018 and has several clinical trials including the surpass intracranial aneurysms embolization system pivotal trial (SCENT) [25] and systematic reviews to support its use [47]. Therefore, some researchers and clinicians may anticipate that the evidence to support SS-FD is sufficient to use the new SE-FD or its use might be based on anecdotal evidence. As with SS-FD, other flow diverters have also been brought into use with strong interventional studies. For example, Pipeline FD was introduced and approved in 2011 [14]. By 2013, the effectiveness and principles of its use to treat brain aneurysm were supported by a well-established clinical trial [39] and several other published clinical studies [27,48].

## 5. Conclusions

The findings of the observational studies included in this review suggest that the SE-FD rate of complete occlusion and clinical and radiological success are lower than in previous versions of FDS. It is suggested that the risk of complications during and after the SE-FD procedure is not higher than the risks in previous FDS. The included studies recommended that SE-FD demonstrates navigability and resistance to twisting which makes the procedure easier to perform when treating aneurysms that are proximal and distal to the circle of Willis deployment. However, we are unable to recommend these suggestions in the absence of clinical trials. Therefore, this review highlights the urgency to conduct clinical trials investigating the effectiveness and safety of SE-FD.

## Figures and Tables

**Table 1:** Quality assessment of included studies.

Items for Case Series Design	Jee et al. (2021) [21]	Lee et al. (2021) [22]	Maus et al. (2021) [23]	Orru et al. (2020) [16]	Rautio et al. (2021) [15]
1. Does the patient(s) represent(s) the whole experience of the investigator (center) or is the selection method unclear to the extent that other patients with similar presentation may not have been reported?		✵	✵	✵	✵
2. Was the exposure adequately ascertained?		✵	✵	✵	✵
3. Was the outcome adequately ascertained?		✵	✵	✵	✵
4. Were other alternative causes that may explain the observation ruled out?		✵	✵	?	✵
5. Is the case(s) described with sufficient details to allow other investigators to replicate the research or to allow practitioners make inferences related to their own practice?		✵	✵	✵	✵
Items for cohort design					
1. Did the study address a clearly focused issue?	✵				
2. Was the cohort recruited in an acceptable way?	✵				
3. Was the exposure accurately measured to minimize bias?	✵				
4. Was the outcome accurately measured to minimize bias?	✵				
5. Have the authors identified all important confounding factors?	?				
6. Have they taken account of the confounding factors in the design and/or analysis?	?				
7. Was the follow up of subjects complete enough?	?				
8. Was the follow up of subjects long enough?	✵				
9. Do you believe the results?	✵				
10. Can the results be applied to the local population?	✵				
11. Do the results of this study fit with other available evidence?	✵				
12. What are the implications of this study for practice?	?				
Total stars out of 12	8/12	5/5	5/5	4/5	5/5

✵ given star; ? missing star

**Table 2:** Included studies’ characteristics.

References	Research Methods	Country of Study	Population	Sample Size	No. of Aneurysms/No. of SE	Participants’ Age Mean/Range	Participants (M/F)
Jee et al. (2021) [21]	Retrospective Cohort	Republic of Korea	Group 1 (SE-FD): patients with intracranial aneurysms treated with SE between June 2019 and December 2020 Group 2 (PED-FD and SS-FD): patients with intracranial aneurysms treated with other FDs (PED-FD and SS-FD) between July 2014 and December 2020	84	84/31	57.5 ± 13.9 Years	Group 1: 16M/15F Group 2: 26M/27F
Lee et al. (2021) [22]	Retrospective Case Series	Republic of Korea	Patients with unruptured VADAs between March 2013 and October 2020 were treated with FDs	12	12/5	54.6/(42–77) Years	9M/3F
Maus et al. (2021) [23]	Retrospective Case series	Germany	Patients with intracranial aneurysms treated with SE between May 2019 to June 2020	42	46/57	58/(28–84) Years	10M/32F
Orru et al. (2020) [16]	Prospective Case series	Canada	Adults with anterior and posterior circulation aneurysms using SE between April and October 2019	25	26/29	58/(36–86) Years	5M/20F
Rautio et al. (2021) [15]	Retrospective Case series	Finland	Adults with intracranial aneurysms (24 unruptured aneurysms, 5 ruptured aneurysms) from May 2019 to January 2020	29	30/31	55.5/(32–72) Years	8M/21F

**Table 3:** Included studies aims, outcomes, and conclusions.

References	Study Main Aims	Primary Outcome	Secondary Outcome	Conclusion
Jee et al. (2021) [21]	Feasibility and safety profile in comparison with a control group treated with other types of flow diverters.	Technical failures, major complications, and unfavorable functional outcomes within 6 months after flow diversion	Procedural time, balloon angioplasty, and diffusion-weighted imaging (DWI)-positive lesions on post-procedural MRI	SE is safe and easy to deploy. However, a study on the long-term safety and efficacy outcomes is required for this new device.
Lee et al. (2021) [22]	Evaluate the outcomes of FDD in large VADAs and assess the safety and feasibility of FDD in the treatment of unruptured large VADAs.	Clinical outcome of last follow-up using modified Rankin Scale (mRS)	Radiographic outcome immediately and 6 months after the procedure using OKM grade	Treatment of large VADAs using FDD is feasible and effective based on the favorable occlusion rate and clinical outcome.
Maus et al. (2021) [23]	Examine the feasibility, efficacy, and safety profile of the new SE flow diverter in the treatment of intracranial wide-necked aneurysms.	Technical success: favorable navigation to the target vessel and successful deployment of the SE	Favorable aneurysm occlusion is defined as OKM grade on follow-up, procedure-related complications, and retreatment	SE flow diverter is safe and effective with promising occlusion rates at short-term follow-up.
Orru et al. (2020) [16]	Describe the results in patients treated with SE.	Immediate post-procedure aneurysm thrombosis using OKM grade	Radiological follow-up, clinical status using mRS, and neurological complications	Demonstrated excellent success rate, good safety, and efficacy of the SE with excellent navigability and resistance to twisting while maintaining high flow diverting effect and positioning.
Rautio et al. (2021) [15]	Safety and six-month follow-up outcomes using the new SE flow diverter in the treatment of intracranial aneurysms.	Clinical safety was assessed by the absence of death, absence of major and minor stroke, and absence of a transient ischemic attack	Treatment efficacy by angiographic occlusion using the OKM grading scale immediately after the procedure and at 6 months follow-up	SE works well with no intraprocedural thromboembolic complications and occlusion rates comparable to other FDs.

**Table 4:** Included studies type of aneurysms, period of follow-up, and description of the procedure.

References	Type of Aneurysms	Type of Aneurysms Treated with SE	Period of Follow up	Description of Procedure
Jee et al. (2021) [21]	Group 1 Location: Anterior circulation: *n* = 20 (64.5%) Posterior circulation: *n* = 11 (35.5%) Size: Mean diameter 18.4 ± 7.6 mm Small *n* = 4 (12.9%) Large *n* = 17 (54.8%) Giant *n* = 10 (32.3%) Shape: Saccular *n* = 11 (35.5%) Non-saccular *n* = 20 (64.5%) Group 2 Location: Anterior circulation: *n* = 35 (66.1%) Posterior circulation: *n* = 18 (34%) Size: Mean diameter 20.6 ± 7.0 mm Small *n* = 0 (0.0%) Large *n* = 37 (69.8%) Giant *n* = 16 (30.2%) Shape: Saccular *n* = 25 (47.2%) Non-saccular *n* = 28 (52.8%)	Location: Anterior circulation: *n* = 20 (64.5%) Posterior circulation: *n* = 11 (35.5%) Size: Mean diameter 18.4 ± 7.6 mm Small *n* = 4 (12.9%) Large *n* = 17 (54.8%) Giant *n* = 10 (32.3%) Shape: Non-saccular *n* = 20 (64.5%) Saccular *n* = 11 (35.5%)	Six months	DAPT aspirin 100 mg + clopidogrel 75 mg for 5–14 days OR loading dose (300 mg) aspirin + clopidogrel for 1 or 2 days given before procedure. Platelet function test was assessed in all patients using VerifyNow Assay. Poor responders were switched to ticlopidine 250 mg twice a day. Specific flow diverter selection was based on the operator’s preference. Immediate and post-flow diversion angiography, as well as, Dyna CT imaging performed in all cases. Balloon angioplasty was performed to improve vessel wall apposition if necessary. Incomplete coverage of target aneurysm neck resulted in additional stenting.
Lee et al. (2021) [22]	Location: Posterior circulation: *n* = 12 -At level PICA *n* = 4 -Proximal to PICA *n* = 4 -Distal to PICA *n* = 4 Size: Large *n* = 11 Giant *n* = 1 Shape: All dissecting aneurysms	Location: Posterior circulation: *n* = 5 -At level PICA *n* = 1 -Proximal to PICA *n* = 2 -Distal to PICA *n* = 2 Size: Large *n* = 5 Shape: All dissecting aneurysms	6–16 months	DAPT aspirin 100 mg + clopidogrel 75 mg daily at least 5 days before the procedure and heparin during the procedure. Platelet function tests were assessed using VerifyNow Assay. The FDD was accessed through the femoral artery. No additional coiling was used. The size of FDD was determined based on the diameter of the parent artery and the length of dissecting segment. VasoCT was performed to confirm wall opposition. DAPT continued for 6 months post-op then switched to aspirin alone.
Maus et al. (2021) [23]	Location: Anterior circulation: *n* = 41 (89%) Posterior circulation: *n* = 5 (11%) Size: Median size 6.6 mm (IQR 4.0–12.2 mm) Median neck width 4 mm (IQR 2.2–5.4 mm) Small *n* = 34 (74%) Large/Giant *n* = 12 (26%) Shape: Saccular *n* = 30 (65%) Fusiform *n* = 10 (22%) Blister *n* = 4 (9%) Dissecting *n* = 2 (4%)	Location: Anterior circulation: *n* = 41 (89%) Posterior circulation: *n* = 5 (11%) Size: Median aneurysm size 6.6 mm (IQR 4.0–12.2 mm) Median neck width 4 mm (IQR 2.2–5.4 mm) Small *n* = 34 (74%) Large/Giant *n* = 12 (26%) Shape: Saccular *n* = 30 (65%) Fusiform *n* = 10 (22%) Blister *n* = 4 (9%) Dissecting *n* = 2 (4%)	Follow-up median 116 days/IQR 92–134 days	DAPT aspirin 100 mg + clopidogrel 75 mg for 5 days prior to procedure. Platelet function test assessed using Multiplate Analyzer. Poor responders either had dose escalation of clopidogrel 150 mg/day or switched to prasugrel. Bolus heparin was administered during the procedure. The FDD was accessed through the femoral artery. The number flow diverter was deployed based on the operator’s discretion. CT scan was performed to confirm aneurysm occlusion. DAPT continued for 3 months post-op then switched to aspirin alone or life.
Orru et al. (2020) [16]	Location: Anterior circulation *n* = 25 (96%) Posterior circulation *n* = 1 (4%) Size: Mean diameter 11mm (range 3–30 mm) Small *n* = 16 (58%) Large *n* = 8 (31%) Giant *n* = 2 (8%) Shape: Saccular *n* = 24 (92%) Fusiform *n* = 1 (4%) Dissecting *n* = 1 (4%)	Location: Anterior circulation *n* = 25 (96%) Posterior circulation *n* = 1 (4%) Size: Mean diameter 11mm (range 3–30 mm) Small *n* = 16 (58%) Large *n* = 8 (31%) Giant *n* = 2 (8%) Fusiform partially thrombosed *n* = 1 (4%) Shape: Saccular *n* = 24 (92%) Fusiform *n* = 1 (4%) Dissecting *n* = 1 (4%)	Five days–six months	DAPT aspirin + ticagrelor/clopidogrel for 3 days before the procedure and continued for at least 6 months. Intravenous heparin was administered intraoperatively. The FD was accessed through the femoral artery or radial artery. Additional FD implanted when indicated and adjunctive coils placed upon operator preference. VasoCT was performed to confirm correct FD wall opposition.
Rautio et al. (2021) [15]	Location: Anterior circulation *n* = 24 Posterior circulation *n* = 6 Size: Small *n* = 20 Large *n* = 9 Giant *n* = 1 Shape: Saccular *n* = 28 Fusiform *n* = 2	Location: Anterior circulation *n* = 24 Posterior circulation *n* = 6 Size: Small *n* = 20 Large *n* = 9 Giant *n* = 1 Shape: Saccular *n* = 28 Fusiform *n* = 2	3–6 months	DAPT aspirin 100 mg + prasugrel 10 mg or clopidogrel 75 mg for at least 5–7 days preoperatively. Platelet function test assessed using Multiplate Analyzer or VerifyNow in elective cases. Bolus heparin was administered intraoperatively. Acute cases were given intravenous 250–500 mg aspirin before, during, and after the procedure with a prasugrel loading dose. FD was accessed through the femoral artery or radial artery. Adjunctive coils are placed upon the operator’s preference. The size of FD was determined based on the diameter of the artery and the main operator decision. VasoCT/DSA imaging was performed to confirm wall opposition.

DAPT: Dual antiplatelet therapy; CT: computerized tomography; PICA: posterior inferior cerebellar artery; FDD: Flow diverter devices; FD: Flow diverter; IQR: Interquartile range; DSA: digital subtraction angiography.

**Table 5:** SE-FDs outcomes.

References	Complete/Partial Occlusion Good Clinical Outcome	mRS/Period	Technical Success	Radiological Outcome
Jee et al. (2021) [21]	Complete occlusion *n* = 11 (35%)/Partial occlusion NR at 6-month follow-up.	NR for all patients	90.3%	Postprocedural MRI within 5 days of flow diversion. DSA, MRI, and/or CT angiography, at 6 months after flow diversion.
Lee et al. (2021) [22]	Complete occlusion *n* = 3 (60%)/Partial occlusion *n* = 2 (40%) at 6 months follow up	mRS ≤ 2/15.8 months	NR	Immediately: OKM B3 *n* = 3 OKM A3 *n* = 2 Follow up: OKM C3 *n* = 2 OKM D *n* = 3
Maus et al. (2021) [23]	Complete occlusion *n* = 27 (75%)/Partial occlusion *n* = 4 (11%) at median follow-up was 116 days.	mRS ≤ 2/at discharge	96%	Follow up: OKM D *n* = 27 OKM C1–C3 *n* = 4 OKM B1 & B3 *n* = 3 OKM A2 *n* = 2
Orru et al. (2020) [16]	Complete occlusion *n* = 13/23 (57%) /Partial occlusion *n* = 9/23 (39%) at 4 months follow up	mRS ≤ 2/at discharge	Excellent technical success rates in all cases	Immediately: OKM D *n* = 1 OKM B *n* = 12 OKM A *n* = 13 (12 of these lesions OKM grade = A3)
Rautio et al. (2021) [15]	Immediate post-op occlusion: Complete occlusion *n* = 0 (0%)/Partial occlusion *n* = 3 (10%) Follow-up 6 months: Complete occlusion 15/27 (56%)/Partial occlusion *n* = 6/27 (22%)	mRS ≤ 2/NR	Encouraging technical success and Good radiological outcomes	Immediately: OKM D *n* = 0/30 OKM C *n* = 3/30 OKM B *n* = 7/30 OKM A *n* = 20/30 Follow up: OKM D *n* = 15/27 OKM C *n* = 6/27 OKM B *n* = 6/27 OKM A *n* = 0/27

NR: Not Reported; mRS: modified Rankin Scale; ASA: anterior spinal artery; OKM: O’Kelly Marotta; ICH: intracerebral hemorrhage.

**Table 6:** SE-FDs complications.

References	Perioperative Complications	Postoperative Complications	Long-Term Morbidity (*n*)	Deaths (*n*)/days Post-Procedure
Jee et al. (2021) [21]	Incomplete wall opposition *n* = 1 (3.2%)—Large Stent migration *n* = 1 (3.2%)—Giant Aneurysm rupture NR	Delayed stent migration *n* = 1 (3.2%) Stent thrombosis *n* = 1 (3.2%) Delayed aneurysm rupture *n* = 1 (3.2%) Ischemic stroke *n* = 1 (3.2%) RIPH Hemorrhage *n* = 0 (0%) Neurological *n* = 0 (0%) Intimal hyperplasia NR	0 long-term morbidity	1 death/16 days
Lee et al. (2021) [22]	No intraprocedural complications	Delayed stent migration NR Stent thrombosis *n* = 0 (0%) Delayed aneurysm rupture *n* = 0 (0%) Ischemic stroke *n* = 0 (0%) RIPH Hemorrhage *n* = 0 (0%) ASA obstruction *n* = 0 (0%) Neurological *n* = 0 (0%) Intimal hyperplasia *n* = 2 (40%)	0 long-term morbidity	0/0
Maus et al. (2021) [23]	Incomplete wall opposition *n* = 1 (2%) Stent migration *n* = 1 (2%) Stent thrombosis *n* = 1 (2%) Aneurysm rupture *n* = 0 (0%)	Delayed stent migration NR Stent thrombosis *n* = 1 (2%) Delayed aneurysm rupture *n* = 0 (0%) Ischemic stroke *n* = 3 (7%) RIPH Hemorrhage *n* = 0 (0%) Neurological (minor) *n* = 1 (2%) Intimal hyperplasia (mild) *n* = 3/34 (9%) (Severe) *n* = 1/34 (3%)	0 long-term morbidity	1 death/10 days
Orru et al. (2020) [16]	No intraprocedural complications	Delayed stent migration NR Stent thrombosis *n* = 1 (4%) Delayed aneurysm rupture NR Ischemic stroke *n* = 1 (4%) RIPH Hemorrhage *n* = 0 (0%) Neurological (minor) *n* = 5 (20%) (Major) *n* = 1 (4%) Intimal hyperplasia NR Others *n* = 3 (12%)	mRS score 4 in one patient suffered a left-sided hemispheric stroke	0/0
Rautio et al. (2021) [15]	No intraprocedural complications	Delayed stent migration NR Stent thrombosis *n* = 2 (7%) Delayed aneurysm rupture *n* = 1 (3%)—Large Ischemic stroke *n* = 1 (3%)—Large RIPH Hemorrhage *n* = 2 (7%) (1 SAH & SAH + ICH)—1 Small, 1 Large Neurological (minor) *n* = 1 (3%) (Major) *n* = 1 (3%) Intimal hyperplasia (minor) *n* = 11 (45%)	One patient’s mRS status changed from 1 to 2 after SAH	2 deaths/6 and 12 days

IVH: intraventricular hemorrhage; mRS: modified Rankin Scale; SAH: subarachnoid hemorrhage; NR: Not Reported; ICH: intracranial hemorrhage, RIPH: remote intraparenchymal hemorrhage.

## Data Availability

Not applicable.

## Supplementary Materials

The following supporting information can be downloaded at: https://www.mdpi.com/article/10.3390/brainsci12060810/s1, S1: Example of MEDLINE search; S2: Case series and cohort studies quality assessment tools.
