# Supplementary material for: Surpass Evolve Flow Diverter for the Treatment of Intracranial Aneurysm: A Systematic Review
Source: Brain Sci. 2022 Jun 20;12(6):810. doi: 10.3390/brainsci12060810 (PMC9221455; doi:10.3390/brainsci12060810)
Supplement: Supplementary file 1 [file brainsci-12-00810-s001.zip › brainsci-1764555-supplementary.pdf]

S1: Example of MEDLINE search

Search	Query	Results
#4	Search: ((Intracranial Aneurysm[MeSH Terms]) OR ("intracranial aneurysm" OR "intracranial aneurysms" OR "brain aneurysm" OR "brain aneurysms" OR "cerebral aneurysm" OR "cerebral aneurysms" OR "unruptured aneurysm" OR "unruptured aneurysms")) AND ("Surpass evolve flow diverter" OR "surpass evolve" OR "Evolve flow diverter" OR "flow diverter") Filters: from 2019/1/1 - 3000/12/12 Sort by: Most Recent (("intracranial aneurysm"[MeSH Terms] OR ("intracranial aneurysm"[All Fields] OR "intracranial aneurysms"[All Fields] OR "brain aneurysm"[All Fields] OR "brain aneurysms"[All Fields] OR "cerebral aneurysm"[All Fields] OR "cerebral aneurysms"[All Fields] OR "unruptured aneurysm"[All Fields] OR "unruptured aneurysms"[All Fields])) AND ("Surpass evolve flow diverter"[All Fields] OR "surpass evolve"[All Fields] OR "Evolve flow diverter"[All Fields] OR "flow diverter"[All Fields])) AND (2019/1/1:3000/12/12[pdat])<br>Translations<br>Intracranial Aneurysm[MeSH Terms]: "intracranial aneurysm"[MeSH Terms]	444
#3	Search: ((Intracranial Aneurysm[MeSH Terms]) OR ("intracranial aneurysm" OR "intracranial aneurysms" OR "brain aneurysm" OR "brain aneurysms" OR "cerebral aneurysm" OR "cerebral aneurysms" OR "unruptured aneurysm" OR "unruptured aneurysms")) AND ("Surpass evolve flow diverter" OR "surpass evolve" OR "Evolve flow diverter" OR "flow diverter") Sort by: Most Recent	1,032
#2	Search: "Surpass evolve flow diverter" OR "surpass evolve" OR "Evolve flow diverter" OR "flow diverter" Sort by: Most Recent	1,220
#1	Search: (Intracranial Aneurysm[MeSH Terms]) OR ("intracranial aneurysm" OR "intracranial aneurysms" OR "brain aneurysm" OR "brain aneurysms" OR "cerebral aneurysm" OR "cerebral aneurysms" OR "unruptured aneurysm" OR "unruptured aneurysms") Sort by: Most Recent	35,422

S2: Case series and cohort studies quality assessment tools

**Case series**		
Reviewer Name:	Date of the review:	
Authors/Year of publication		
**Does the patient(s) represent(s) the whole experience of the investigator (centre) or is the selection method unclear to the extent that other patients with similar presentation may not have been reported?**		
	YES ✸<br>Can’t tell<br>NO	
**Was the exposure adequately ascertained?**		
	YES ✸<br>Can’t tell<br>NO	
**Was the outcome adequately ascertained?**		
	YES✸<br>Can’t tell<br>NO	
**Were other alternative causes that may explain the observation ruled out?**		

	YES ✵<br>Can’t tell<br>NO	
**Was there a challenge/rechallenge phenomenon?**		
	YES ✵<br>Can’t tell<br>NO	
**Was there a dose–response effect?**		
	YES ✵<br>Can’t tell<br>NO	
**Was follow-up long enough for outcomes to occur?**		
	YES ✵<br>Can’t tell<br>NO	
**Is the case(s) described with sufficient details to allow other investigators to replicate the research or to allow practitioners make inferences related to their own practice?**		
	YES ✵<br>Can’t tell<br>NO	
**Total number of stars (Maximum of 8 stars)**		

**Cohort**		
Reviewer Name:	Date of the review:	
Authors/Year of publication		
**Did the study address a clearly focused issue?**		
HINT: A question can be ‘focused’ in terms of • the population studied • the risk factors studied • is it clear whether the study tried to detect a beneficial or harmful effect • the outcomes considered	YES ✵<br>Can’t tell<br>NO	
**Was the cohort recruited in an acceptable way?**		
HINT: Look for selection bias which might compromise the generalisability of the findings: • was the cohort representative of a defined population • was there something special about the cohort • was everybody included who should have been	YES ✵<br>Can’t tell<br>NO	
**Was the exposure accurately measured to minimise bias?**		
HINT: Look for measurement or classification bias: • did they use subjective or objective measurements • do the measurements truly reflect what you want them to (have they been validated) • were all the subjects classified into exposure groups using the same procedure	YES✵<br>Can’t tell<br>NO	
**Was the outcome accurately measured to minimise bias?**		
HINT: Look for measurement or classification bias: • did they use subjective or objective measurements • do the measurements truly reflect what you want them to (have they been validated) • has a reliable system been established for detecting all the cases (for measuring disease occurrence) • were the measurement methods similar in the different groups • were the subjects and/or the outcome assessor blinded to exposure (does this matter)	YES ✵<br>Can’t tell<br>NO	
**Have the authors identified all important confounding factors?**		
HINT: • list the ones you think might be important, and ones the author missed	YES ✵<br>Can’t tell<br>NO	
**Have they taken account of the confounding factors in the design and/or analysis?**		

HINT: • look for restriction in design, and techniques e.g. modelling, stratified-, regression-, or sensitivity analysis to correct, control or adjust for confounding factors	YES ✵<br>Can't tell<br>NO	
**Was the follow up of subjects complete enough?**		
HINT: Consider • the good or bad effects should have had long enough to reveal themselves • the persons that are lost to follow-up may have different outcomes than those available for assessment • in an open or dynamic cohort, was there anything special about the outcome of the people leaving, or the exposure of the people entering the cohort	YES ✵<br>Can't tell<br>NO	
**Was the follow up of subjects long enough?**		
	YES ✵<br>Can't tell<br>NO	
**Do you believe the results?**		
HINT: Consider • big effect is hard to ignore • can it be due to bias, chance or confounding • are the design and methods of this study sufficiently flawed to make the results unreliable • Bradford Hills criteria (e.g. time sequence, dose-response gradient, biological plausibility, consistency)	YES ✵<br>Can't tell<br>NO	
**Can the results be applied to the local population?**		
HINT: Consider whether • a cohort study was the appropriate method to answer this question • the subjects covered in this study could be sufficiently different from your population to cause concern • your local setting is likely to differ much from that of the study • you can quantify the local benefits and harms	YES ✵<br>Can't tell<br>NO	
**Do the results of this study fit with other available evidence?**		
	YES ✵<br>Can't tell<br>NO	
**What are the implications of this study for practice?**		
HINT: Consider • one observational study rarely provides sufficiently robust evidence to recommend changes to clinical practice or within health policy decision making • for certain questions, observational studies provide the only evidence • recommendations from observational studies are always stronger when supported by other evidence	YES ✵<br>Can't tell<br>NO	
**Total number of stars (Maximum of 12 stars)**		
